# Predictive Factors for Adverse Cardiac Events and Mortality in Patients with Hypertrophic Cardiomyopathy

**DOI:** 10.3390/jcm14103546

**Published:** 2025-05-19

**Authors:** Hazem Omran, Tanja K. Rudolph, Lothar Faber, Volker Rudolph, Zisis Dimitriadis

**Affiliations:** 1 Clinic for General and Interventional Cardiology/Angiology, Herz- und Diabeteszentrum NRW, Ruhr-Universität Bochum, 32545 Bad Oeynhausen, Germany; trudolph@hdz-nrw.de (T.K.R.); vrudolph@hdz-nrw.de (V.R.); 2 Medical Clinic II, Lukas-Hospital, 32257 Bünde, Germany; faber-lothar@t-online.de; 3 Department of Cardiology I, Universitätsmedizin Mainz und DZHK Standort Rhein-Main, 55131 Mainz, Germany; dimitriadis.zisis@gmail.com

**Keywords:** hypertrophic cardiomyopathy, sudden cardiac death, implantable cardioverter defibrillator, risk stratification

## Abstract

**Background/Objectives:** Risk stratification for sudden cardiac death (SCD) in hypertrophic cardiomyopathy (HCM) remains challenging, especially in high-risk cohorts. This study evaluated the predictive utility of the ESC HCM Risk Score and the additive value of myocardial fibrosis assessment via cardiac magnetic resonance (CMR) in HCM patients with implantable cardioverter-defibrillators (ICDs) for primary prevention. **Methods**: A retrospective analysis was conducted on 108 HCM patients (mean age 49.4 ± 14.2 years; 30.6% female; 63.9% with LVOT obstruction) with ICDs for primary SCD prevention. The primary endpoint was a composite of all-cause mortality or appropriate ICD therapy for ventricular arrhythmia over a mean follow-up of 69.5 ± 22.8 months. ESC HCM Risk Scores, the presence of fibrosis on CMR, and clinical outcomes were analyzed using univariate and multivariate models, ROC curves, and Kaplan–Meier survival estimates. **Results:** The primary endpoint occurred in 25 patients (23.1%; 3.1%/year). An ESC HCM Risk Score ≥ 4% was common (81.5%) but did not significantly predict the primary outcome (the c-statistic 0.54; *p* = 0.08) and demonstrated low positive (25%) and high negative predictive values (85%). Severe fibrosis on CMR was significantly associated with events in univariate analysis (*p* = 0.04), and its inclusion improved the model’s predictive accuracy (the c-statistic increased to 0.65; *p* = 0.03). Kaplan–Meier analysis revealed worse event-free survival in patients with both elevated ESC scores and more than mild fibrosis (*p* = 0.028). **Conclusions**: In this high-risk HCM cohort with ICDs, the ESC risk score showed limited predictive performance, while myocardial fibrosis on CMR added significant prognostic value. Incorporating the fibrosis assessment into future risk models may enhance SCD prediction and refine ICD decision-making in HCM. Further multicenter studies are needed to validate these findings.

## 1. Background

Hypertrophic cardiomyopathy (HCM) is one of the leading causes of sudden cardiac death (SCD) in younger people and athletes [1]. The implantable cardioverter-defibrillator (ICD) is an effective preventive therapy in reducing SCD in selected patients at higher risk [1]. Therefore, identifying risk factors for SCD to guide prevention strategies is of great significance. In 2011, the American College of Cardiology Foundation (ACCF)/American Heart Association (AHA) published clinical practice guidelines to assess the risk factors for SCD among patients with HCM and proposed five established clinical risk factors [2], including a family history of SCD in first-degree relatives; an unexplained syncope; non-sustained ventricular tachycardia (nsVT); a maximum left ventricular wall thickness (LVWT) of >30 mm; and an abnormal blood pressure response during exercise. The cumulative positive predictive value of this model was low (approximately 20%) but with a relatively high negative predictive value of approximately 95% [3]. In 2014, the European Society of Cardiology (ESC) introduced a risk prediction model that estimated the 5-year risk of sudden cardiac death (SCD) in patients with hypertrophic cardiomyopathy (HCM) [1]. This model incorporated several newly identified risk factors, such as age, left atrial (LA) diameter, and left ventricular outflow tract obstruction (LVOTO), but did not include the late gadolinium enhancement (LGE) seen on cardiac magnetic resonance imaging (CMR). The 2024 AHA/ACCF guidelines later expanded the model to include additional predictors, such as LGE on CMR, reduced left ventricular ejection fraction (LVEF), and left ventricular apical aneurysm [4,5]. While the 2014 ESC guidelines acknowledged that the extent of LGE could help predict cardiovascular mortality, the evidence at the time was insufficient to support its use in assessing SCD risk [1]. More recent research has shown that LGE is detected in approximately 65% of patients with HCM, typically presenting as patchy mid-wall fibrosis in hypertrophied segments and at the right ventricular insertion points. In non-hypertrophied myocardium, LGE is uncommon except in advanced disease, where it often appears as transmural enhancement with associated wall thinning. The presence of LGE correlates with increased myocardial stiffness, adverse left ventricular remodeling, and a higher incidence of regional wall motion abnormalities. Although quantification methods vary, the two-standard deviation technique remains the only approach validated against histopathology, and extensive LGE (>15% of left ventricular mass) is linked to a higher risk of SCD and other adverse outcomes [5,6,7]. Nonetheless, the most recent ESC guidelines continue to recommend using the HCM-SCD Risk Calculator as the primary tool for risk assessment. In cases where patients fall into the low-to-intermediate risk category, the presence of significant LGE (≥15%) may be considered during shared decision-making about preventive ICD implantation [6]. According to the ESC guidelines, ICD implantation for the primary prevention of SCD should be considered if the calculated 5-year SCD risk is ≥6% (Class IIa) and may be considered if the calculated 5-year SCD risk is between 4 and 6% (Class IIb) [1,6].

The 2003 ACC/ESC guidelines recommended ICD implantation for the primary prevention of SCD if at least two conventional risk factors were positive [8]. And the 2011 ACCF/AHA guidelines used a similar approach. However, an ICD was also recommended if only one of the following risk factors was positive: a family history of SCD, a maximal LV-septal thickness of ≥30 mm, or an unexplained syncope [2]. The latest AHA/ACCF guidelines recommended an ICD if at least one of the following was positive: a family history of SCD, massive LV hypertrophy, a recent unexplained syncope, LVEF < 50% or apical aneurysm, nsVT on Holter ECG, and extensive LGE on CMR [4]. Despite efforts to identify risk factors and calculate SCD probabilities, reliable prediction remains a challenge. The low overall incidence of sudden cardiac death makes risk prediction difficult and results in low positive predictive values. A further problem in applying guideline recommendations is the inconsistency of the results when different models are applied to one patient. This could lead to an inappropriately high rate of ICD implantations, particularly in borderline cases. One study reported that only 18% of HCM patients with ICD received appropriate therapy, while 20% of them encountered inappropriate shocks [9]. Therefore, it seems compulsory to search for new risk factors for SCD in patients with HCM. The aim of this study was to retrospectively assess the utility of guideline recommendations, especially the ESC risk calculator, in risk prediction and to evaluate the significance of individual risk markers in patients with HCM.

## 2. Methods

We performed a retrospective analysis of consecutive patients with HCM treated at our hospital between 1 January 2005 and 1 January 2013 who had received an implantable cardioverter-defibrillator (ICD) for the prevention of sudden cardiac death (SCD). We included only patients with an ICD to ensure adequate follow-ups for all patients. We excluded patients <16 years old and those with HCM secondary to Fabry’s disease, amyloidosis, mitochondria, or other forms of congenital storage heart disease.

### 2.1. Echocardiography

Baseline echocardiographic assessments were performed using 2D imaging (Vivid 7™, GE Healthcare, Wiesbaden, Germany) in accordance with American Society of Echocardiography guidelines [10]. Left atrial (LA) dimensions and left ventricular wall thickness (LVWT) were measured, and left ventricular ejection fraction (LVEF) was calculated using the modified Simpson’s method. Left ventricular outflow tract obstruction (LVOTO) and the systolic anterior motion of the mitral valve were evaluated, with outflow gradients assessed at rest and during provocation using continuous-wave Doppler. The Valsalva maneuver was utilized as the standard modality for provocation to induce an obstruction on the echocardiography in all patients. All studies were reviewed by a single, blinded echocardiographer.

### 2.2. Cardiopulmonary Exercise Testing (CPET)

Symptom-limited bicycle CPET was conducted using an incremental protocol (10 W/min; ZAN Ferraris, Oberthulba, Germany). Peak oxygen uptake (VO_2_ peak), maximum workload, and total exercise time were recorded. The predicted VO_2_ peak was automatically calculated based on age and sex.

### 2.3. Cardiac Magnetic Resonance Imaging (CMR)

CMR was performed on a 3T scanner (Philips Achieva, Best, The Netherlands) with ECG gating and breath-holding sequences. LGE was evaluated using the AHA 17-segment model and scored semi-quantitatively (0–3 scale: absent, mild, moderate, and severe) by an independent, blinded expert. All CMR studies were performed before ICD Implantation.

### 2.4. ICD Interrogation

All patients were implanted with ICD Devices that were conditionally compatible with CMR (up to 1.5 Tesla) in order to allow further follow-up studies. ICD checks were conducted biannually by independent electrophysiologists. Stored electrograms were reviewed to identify appropriate therapies (ATP or shocks) for sustained or non-sustained ventricular arrhythmias. Interventions were considered inappropriate if triggered by supraventricular tachycardias, self-terminating VT, over-sensing, or lead malfunction.

Follow-up: Patients’ symptoms (NYHA functional class) and events (death) were assessed through routine follow-up visits (every 6 months) or via phone calls and contact with the primary care physicians and events were entered into our hospital registry. Patients had already signed informed consent that allowed for future contact and inquiries.

As we aimed to assess the predictive value of the ESC 2014 risk score calculator [1], we excluded patients who received a secondary preventive ICD. The variables considered in our study included the following: (i) a family history of SCD in ≥1 first-degree relatives aged <40 years or in a first-degree relative with confirmed HCM at any age; (ii) maximal left ventricular wall thickness; (iii) a history of unexplained syncope; (iv) documented non-sustained ventricular tachycardia (NSVT) defined as ≥3 beats at a rate of ≥120 beats/min; (v) an abnormal blood pressure response during exercise; (vi) age at presentation; (vii) a maximal (provocated) left ventricular outflow tract (LVOT) gradient; and (viii) left atrial (LA) diameter measured in the parasternal left axis view. We also assessed other parameters that were considered in previous guidelines like left ventricular hypertrophy with a maximal LV wall thickness (LVWT) of ≥30 mm, the presence of atrial fibrillation (AF), and the presence and magnitude of fibrosis on CMR assessed through late gadolinium enhancement (LGE). LVOT obstruction (LVOTO) was defined in accordance with the ESC guidelines [1] as a peak instantaneous gradient of ≥30 mmHg either at rest or during the Valsalva maneuver.

The 5-year SCD risk was calculated using the HCM SCD risk formula as reported in the guidelines. [1] The primary endpoint of this study was a composite of all-cause mortality or appropriate ICD treatment for ventricular tachyarrhythmia. The most severe endpoint was considered in the final assessment, i.e., if patients had received both anti-tachycardia-pacing (ATP) and ICD shocks, they were considered to have had ICD shocks. And in patients undergoing ICD treatment who consequently died, their death was considered.

The study was approved by our local ethics committee and was performed according to Good Clinical Practice. Due to the retrospective characteristic of this study, informed consent was waived by our ethics committee.

## 3. Statistics

Statistical analyses were conducted using IBM SPSS version 27 (IBM Corp., Armonk, NY, USA). Categorical variables are presented as percentages, while continuous variables are reported as the mean ± standard deviation (SD) or median ± interquartile range (IQR), as appropriate. We compared continuous variables using Student’s *t*-test and Mann–Whitney U test as appropriate. Comparisons of categorical variables between groups were performed using Pearson’s X^2^ test and for expected frequencies <5 using Fisher’s exact test.

For survival analysis, Cox proportional hazards regression was applied to assess predictors of the primary outcome and adjust for potential differences in baseline characteristics. Initially, univariate analyses were conducted, and variables with a *p*-value less than 0.1 were subsequently included in the multivariate analysis. Receiver operating characteristic (ROC) analysis was performed to determine the diagnostic utility of different risk models. Differences in event-free survival according to identified risk models were compared using the Kaplan–Meier method and the Log-Rank test. All *p*-values were two-sided, with statistical significance defined as a *p*-value of 0.05.

## 4. Results

A total of 108 patients with HCM with an ICD for the primary prevention of SCD were included (mean age 49.4 ± 14.2 years; 30.6% females; 63.9% with LVOT-obstruction). Details of baseline characteristics are provided in Table 1. The mean ESC risk score was 8.23 ± 4.89%.

Of note, 88 patients (81.5%) had a risk score of 4% or higher, while in 67 patients (62%), the ESC risk score was 6% or greater. The AHA 2003 criteria [8], as well as the ACCF/AHA 2011 criteria [2], were positive in 96 patients (88.9%). On the other hand, AHA/ACCF 2020/2024 criteria [4,5] were positive in 98 patients (90.6%), as detailed in Table 2.

Over a mean follow-up period of 69.5 ± 22.8 months, the primary endpoint (a composite of all-cause mortality or appropriate ICD treatment for ventricular tachyarrhythmia) occurred in 25 patients (7 patients died; 7 received appropriate ATP; and 11 received appropriate ICD shocks). The rate of appropriate ICD treatment was 3.1%/year.

**Figure 1:**
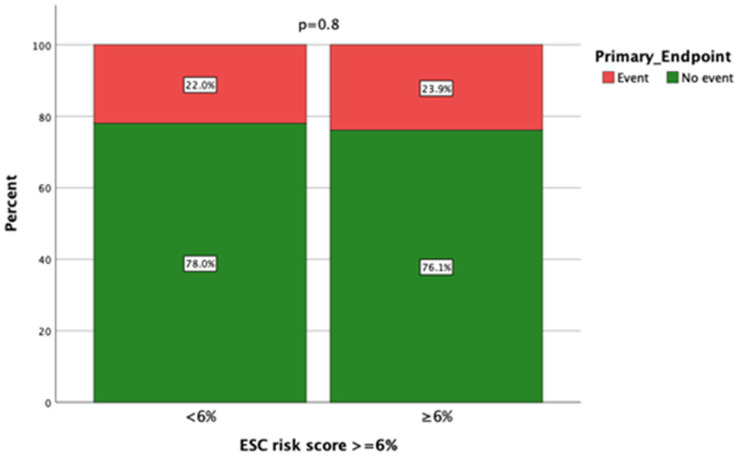
The relationship between the ESC SCD risk score and primary outcome (a composite of all-cause mortality or appropriate ICD treatment for ventricular tachyarrhythmia).

As represented in Table 1, patients who encountered a primary outcome event had greater LA diameters and a more frequently depressed LVEF compared to patients without events. Moreover, severe myocardial fibrosis on CMR was encountered more frequently in patients with primary outcome events (60%) compared to patients without events (36.1%), *p* = 0.04. The mean HCM Risk Score was not significantly different between those with and those without a primary outcome event (Table 1). The primary outcome was also not significantly different between patients with HCM Risk Scores ≥ 4% compared to those with a score <4% (*p* = 0.56). The same applied to a score of >6% (Figure 1). However, most patients (88 out of 108; 81.5%) had an ESC risk score of ≥ 4%, and for those with a low score <4%, the majority (17/20) experienced no primary outcome events over the follow-up period. Correspondingly, the positive predictive value was as low as 25%, but the negative predictive value was rather high at 85%.

Moreover, the HCM Risk Score did not differentiate patients with (7.95 ± 5.0%) vs. without (7.81 ± 4.1%) appropriate ICD treatments (*p* = 0.89), nor were survivors (8.6 ± 4.6%) separated from non-survivors (7.8 ± 4.2%, *p* = 0.61). For multivariable Cox regression analysis (Table 3), the ESC risk score was not a significant predictor of primary outcomes (*p* = 0.08). The finding of severe fibrosis on CMR was significant only in univariate analysis but not in multivariate analysis. Significant predictors of primary outcomes included age, the LA diameter, and maximal LV wall thickness, as represented in Table 3.

**Figure 2:**
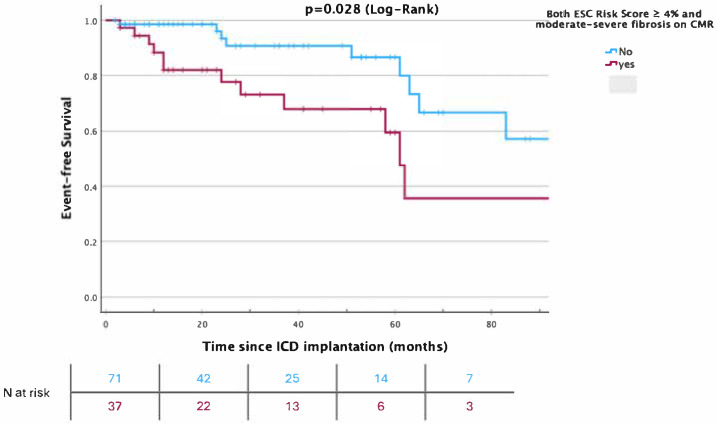
Kaplan–Meier curves for survival free from a primary outcome event (a composite of all-cause mortality or appropriate ICD treatment for ventricular tachyarrhythmia) according to the ESC risk score and more than mild fibrosis on cardiac magnetic resonance imaging (CMR).

Receiver operating characteristic (ROC) analysis revealed a c-statistic of 0.54 and a 95% CI [0.42–0.67] for HCM Risk Scores ≥4% to predict the primary outcome. When severe fibrosis on CMR was added to the predictive model, the c-statistic improved significantly to 0.65, 95%CI [0.52–0.78], *p* = 0.03. Kaplan–Meier survival analysis showed that event-free survival was significantly worse in those with both an ESC risk score ≥ 4% and more than mild fibrosis on CMR (Log-Rank *p* = 0.028), as shown in Figure 2.

## 5. Discussion

In this cohort of 108 HCM patients with an ICD implanted for the primary prevention of sudden cardiac death (SCD), the ESC HCM Risk Score showed limited predictive utility for adverse outcomes. Although 81.5% of patients had a risk score ≥ 4%, and 62% had scores ≥ 6%, the score did not significantly discriminate between patients with and without the primary composite endpoint (all-cause mortality or appropriate ICD therapy), nor between survivors and non-survivors. The overall rate of appropriate ICD interventions was 3.1% per year. Importantly, the ESC risk score demonstrated a low positive predictive value (25%) but a high negative predictive value (85%). Severe myocardial fibrosis on CMR was significantly associated with adverse events in univariate analysis, and adding this parameter to the ESC score improved the model’s discriminative ability (c-statistic from 0.54 to 0.65, *p* = 0.03). Kaplan–Meier analysis further highlighted worse outcomes in patients with both elevated risk scores and more than mild fibrosis. These findings suggest that myocardial fibrosis assessment may enhance risk stratification beyond the established scoring models.

The ESC risk score’s performance revealed a low positive predictive value (25%) but a high negative predictive value (85%). This suggests that while the score may reliably identify patients unlikely to experience adverse events, it struggles to accurately predict which high-risk patients will encounter the endpoint. This pattern is consistent with prior evaluations of the ESC score, which have shown variable discriminatory power. For instance, Vriesendorp et al. reported a c-statistic of 0.61 for SCD prediction [11], while O’Mahony et al., in the score’s original validation, achieved a c-statistic of 0.70 [12]. In contrast, our study’s c-statistic of 0.54 indicates poor discrimination, potentially due to the homogeneity of risk in this cohort, where most patients were already classified as high risk, limiting the score’s ability to stratify further. Moreover, the overall event rate in the study cohort was relatively low, even among patients who received ICDs based on conventional criteria. This finding further underscores the complexity of risk prediction and highlights the potential utility of these models in identifying patients who are unlikely to benefit from ICD implantation.

A key finding was the significant association between severe myocardial fibrosis, assessed via cardiac magnetic resonance (CMR), and adverse events in univariate analysis. Integrating this fibrosis parameter with the ESC score enhanced the model’s discriminative ability, increasing the c-statistic from 0.54 to 0.65 (*p* = 0.03). Kaplan–Meier analysis further underscored the prognostic value of fibrosis, showing worse outcomes in patients with both elevated risk scores and more than mild fibrosis. These results align with a robust body of evidence linking myocardial fibrosis, often detected as late gadolinium enhancement (LGE) on CMR, to increased SCD risk in HCM [13,14]. A meta-analysis by Green et al. reinforced LGE’s potential as a risk stratification tool, though it remained absent from standard models [5,6,15].

Severe fibrosis on CMR has already been reported to be associated with ventricular arrhythmias in several studies, and the AHA guidelines from 2011 and 2020 recommended its use to help with risk stratification. [2,4] Moreover, fibrosis is very frequently found on CMR in patients with HCM, and there is a lack of a standardized assessment for its degree, which is still subjective and examiner-dependent. This could explain why the ESC risk score did not include LGE on CMR for the risk score calculator. Instead, it is an add-on parameter that helps with risk stratification in borderline cases. Our study supports the high prevalence of fibrosis on CMR in patients with HCM and its additive role in the identification of patients with arrhythmic events. The incorporation of a fibrosis assessment offers incremental prognostic value beyond the ESC score alone, suggesting its potential refinement in risk stratification. Clinically, this could guide ICD implantation decisions, particularly in cases where the ESC score is ambiguous. For example, patients with high scores but minimal fibrosis might face a lower risk than anticipated, while those with significant fibrosis may require heightened intervention, regardless of the score.

Further studies are needed to shed more light on this issue.

## 6. Limitations

This study has several limitations. It included a retrospective, single-center analysis of 108 patients referred to our hospital, which may introduce selection bias and limit the generalizability of the findings. Baseline heterogeneity and modest sample sizes, along with a relatively low number of outcome events reduced the statistical power to detect significant predictors of MACE. Follow-up data on ICD therapy were only available for patients who returned to our center, potentially leading to the incomplete collection of data on outcomes. Additionally, the presence of ICDs in all patients may have influenced disease progression, although this was partially addressed by including appropriate ICD therapy in the composite endpoint. The assessment of myocardial fibrosis by CMR was performed subjectively by a single radiologist without external validation, and the definition of “severe” fibrosis was not standardized, reflecting the absence of a uniform grading scheme. A notable limitation of this study is the absence of speckle-tracking echocardiography (STE) data, which may have provided additional insight into subclinical myocardial dysfunction in patients with HCM. STE, particularly through the assessment of the global longitudinal strain (GLS), has been shown to detect early myocardial impairment despite preserved left ventricular ejection fraction [16,17] and to correlate closely with the extent of myocardial fibrosis, as assessed by cardiac MRI [18]. The lack of STE-derived strain measurements in our cohort may limit our ability to fully characterize myocardial functional changes and their association with underlying structural abnormalities. Future studies incorporating STE could enhance the diagnostic and prognostic evaluation of HCM patients.

## 7. Conclusions

In conclusion, the ESC HCM Risk Score exhibited limited predictive power in this high-risk HCM cohort with ICDs, while myocardial fibrosis assessment via CMR provided valuable additional insights. These findings advocate for the integration of imaging-based fibrosis evaluation into future risk models to improve SCD prediction in HCM. Larger, multicenter studies are warranted to confirm these observations and optimize the role of CMR in clinical practice.

## Figures and Tables

**Table 1:** Baseline characteristics.

Variable	Patients with Primary Endpoint	Patients Without Primary Endpoint	Total	*p*-Value
n (%)	25 (23.1%)	83 (76.9%)	108 (100%)	
Age (y)	53.5 ± 13.5	48.2 ± 14.3	49.4 ± 14.2	0.10
BMI kg/m^2^	27.1 ± 3.8	27.6 ± 4.7	27.5 ± 4.5	0.59
Female n (%)	7 (28%)	26 (31.3%)	33 (30.6%)	0.75
HOCM n (%)	12 (48%)	57 (68.7%)	69 (63.9%)	0.06
NYHA	2 (±0.83)	2 (±0.86)	2(±0.85)	0.93
Max. LVWT (mm)	24.4 ± 6.2	25.6 ± 7.2	25.4 ± 7.0	0.42
LA diameter (mm)	54.9 ± 15.5	47.4 ± 7.8	49.1 ± 10.5	0.03
LVEF < 50%	4 (16%)	3 (3.6%)	7 (6.5%)	0.049
Peak VO_2_ (mL/min/kg)	19.7 ± 7.9	19.8 ± 6.8	19.8 ± 7.0	0.96
ESC risk score	8.23 ± 4.89	7.71 ± 4.04	7.83 ± 4.24	0.60
CAD	3 (12%)	8 (9.6%)	11 (10.2%)	0.71
COPD	4 (16%)	6 (7.2%)	10 (9.6%)	0.24
Any fibrosis on CMR	24 (96%)	75 (90.4%)	99 (91.7%)	0.68
More than mild fibrosis on CMR	15 (60%)	30 (36.1%)	45 (42.6%)	0.04
Inappropriate BP on exercise	9 (36%)	24 (29%)	33 (30.6%)	0.50
Any family history for SCD	5 (20%)	24 (29%)	29 (26.9%)	0.38
SCD in 1st-degree relatives	4 (16%)	11 (13.3%)	15 (13.9%)	0.75
HCM in 1st-degree relatives	2 (8%)	5 (6%)	7 (6.5%)	0.66
Unexplained syncope	12 (48%)	31 (37.3%)	43 (39.8%)	0.34
NSVT	16 (64%)	41 (49.4%)	57 (52.8%)	0.20

Table legend: BMI: body mass index; HOCM: hypertrophic obstructive cardiomyopathy; NYHA: New York Heart Association functional class; LVWT: left ventricular wall thickness; LA: left atrium; VO_2_: O_2_ consumption; CAD: coronary artery disease; COPD: chronic obstructive pulmonary disease; CMR: cardiac magnetic resonance imaging; BP: blood pressure; SCD: sudden cardiac death; NSVT: non-sustained ventricular tachycardia.

**Table 2:** Risk assessment models according to primary outcome.

Risk Assessment Model	Patients with Primary Endpoint	Patients Without Primary Endpoint	Total	*p*-Value
ESC risk score	8.23 ± 4.89	7.71 ± 4.04	7.83 ± 4.24	0.60
ESC score ≥ 4%	22 (88%)	66 (79.5%)	88 (81.5%)	0.56
ESC score ≥ 6%	16 (64%)	51 (61.4%)	67 (62%)	0.81
ACCF/AHA 2011 [2]	23 (92%)	73 (88%)	96 (88.9%)	0.73
ACCF/AHA 2024 [5]	25 (100%)	73 (88%)	98 (90.6%)	0.11

**Table 3:** Cox proportional hazards regression analysis of predictors of primary outcomes.

Variable	HR [95% CI] (Univariate)	*p*-Value (Univariate)	HR [95% CI] (Multivariate)	*p*-Value (Multivariate)
Age	1.06 [1.02–1.09]	<0.001	1.09 [1.04–1.14]	0.012
LA diameter	1.04 [1.01–1.06]	0.002	1.05 [1.01–1.08]	0.005
LVEF < 50%	5.07 [1.6–15.6]	0.005	2.9 [0.74–11.4]	0.12
Max. LVWT	1.12 [1.01–1.24]	0.009	1.11 [1.03–1.21]	0.01
Severe fibrosis on CMR	1.03 [1.01–1.05]	0.04	1.05 [0.98–1.15]	0.08
HOCM	0.61 [0.27–1.35]	0.2	0.93 [0.34–2.59]	0.8
Family history SCD (1st degree)	2.5 [0.83–2.74]	0.09	2.1 [0.62–7.25]	0.23
Peak VO_2_	0.94 [0.88–1.0]	0.06	0.98 [0.90–1.1]	0.76
ESC risk score	1.06 [0.99–1.24]	0.09	1.08 [0.99–1.27]	0.08

## Data Availability

The data presented in this study are available on request from the corresponding author due to data protection regulations and medical confidentiality.
